# Indigo—A New Tribological Substance Class for Non-Toxic and Ecological Gliding Surfaces on Ice, Snow, and Water

**DOI:** 10.3390/ma15030883

**Published:** 2022-01-24

**Authors:** Peter Bützer, Dominik Brühwiler, Marcel Roland Bützer, Nassim Al-Godari, Michelle Cadalbert, Mathias Giger, Sandro Schär

**Affiliations:** 1 Isantin GmbH, 9450 Altstätten, Switzerland; m.buetzer@isantin.ch; 2 Institute of Chemistry and Biotechnology, Zürich University of Applied Sciences (ZHAW), 8820 Wädenswil, Switzerland; algodnas@students.zhaw.ch (N.A.-G.); michellecadalbert@hotmail.com (M.C.); gigerma1@students.zhaw.ch (M.G.); schaer.sandro@gmail.com (S.S.)

**Keywords:** indigo, gliding surface, supramolecular, self-assembly, ice, snow, water, QSAR

## Abstract

The biogenic substance E-indigo can form supramolecular, hydrophobic structures using self-organization. These structures show a low coefficient of friction as a gliding layer against polar surfaces. The formation of primary particles with platelet morphology based on hydrogen-bonded E-indigo molecules is ideal to produce the gliding layer. Structures with excellent gliding properties can be achieved by means of directed friction and high pressure, as well as through tempering. The resulting hard, thin gliding layer of E-indigo does not easily absorb dirt and, thus, prevents a rapid increase in friction. Field tests on snow, with cross-country skis, have shown promising results in comparison to fluorinated and non-fluorinated waxes. Based on quantitative structure–activity relationship (QSAR) data for E-indigo, and its isomers and tautomers, it has been demonstrated that both the application and abrasion of the thin indigo layers are harmless to health, and are ecologically benign and, therefore, sustainable.

## 1. Introduction

Archaeological research identified indigo-dyed textiles dating back to at least 7800 years ago [1]. Since that time, indigo has been used as a textile dye on every continent and it is ubiquitous today as the dye used for blue jeans. As an artist’s pigment, indigo, which is insensitive to oxidation, has been found in Roman paintings from the first century AD. Indigo is also well known as the chromophore of the pre-Columbian pigment Maya Blue [2]. The unusual stability of Maya Blue has inspired the use of indigo as a chromophore in other host–guest-based colorants [3].

A limiting factor in the application of indigo is its poor solubility in most solvents, e.g., 1 ppm in water and in the per mil range in solvents such as aniline, chloroform, glacial acetic acid, concentrated sulfuric acid, dichloromethane, dimethyl sulfoxide, nitrobenzene, and pyridine [4], thus rendering chemical analysis difficult. However, the strong interactions between indigo molecules can be exploited for the formation of supramolecular, self-assembled layers [5]. Such layers of indigo feature a metallic luster, which can, for example, be observed on the turbans of the Tuareg [6] and the clothes of the Miao [7]. As a thin film, pure E-indigo and indigo derivatives have been used as biocompatible semiconductor materials in organic field-effect transistors [8,9].

Indigo is safe for human health and is not harmful to the environment, which are essential conditions for wide application [10]. Poorly purified indigo is employed in tattoos [11], and in the context of its use in the textile industry, there have been no traceable negative physiological effects caused by permanent skin contact [12]. In the environment, indigo is a natural metabolic product of plants on all continents, and has no negative specific bioactive effects for concentrations up to 1300 mg/m^2^ [13,14].

Indigoid substances are not known in tribology, but, as shown by our experiments, many of the molecular properties of indigo suggest that thin, hard, hydrophobic layers of areal indigo clusters are suitable as gliding surfaces on ice, snow, and water. Based on promising initial results, this class of indigoid substances was patented for use in winter sports equipment [15].

As early as 1673, greases and oils were described as lubricants for skis [16], which were complemented and replaced over time by resins, waxes, siloxanes, and other additives, with the most successful new developments being waxes based on fluorinated compounds. First patented in 1983 [17], perfluorocarbon lubricants were subsequently marketed with different compositions by various manufacturers. Poly- and per-fluorinated compounds are increasingly subject to restrictions and bans [18,19], which is why, in 2019, the International Ski Federation (FIS) planned a ban on fluorinated ski waxes in competitions [20]. Indigo represents an occupational hygienic and ecologically harmless alternative to fluorinated ski waxes. The use of E-indigo for non-toxic gliding surfaces and the associated challenges are discussed in this review.

## 2. Synthesis and Specific Properties of Indigo

### 2.1. Synthesis of Indigo

**Figure 1:**
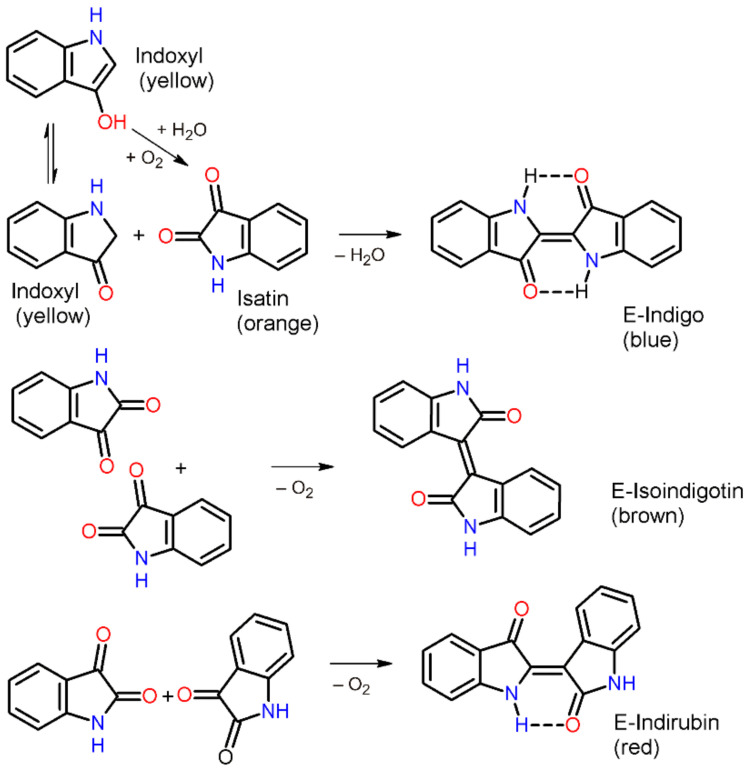
Synthesis of E-indigo, E-isoindigotin and E-indirubin from isatin and indoxyl. Two intramolecular hydrogen bonds can be formed only in the case of the isomer E-indigo.

In the synthesis from plant sap, indoxyl reacts with oxygen and water to form the three isomers indigo blue (indigo), indigo brown (isoindigotin), and indigo red (indirubin), which were already described in plant extracts by Jöns Jakob Berzelius (1779–1848). The chemical synthesis starts from the same reactants and forms the same isomers, whose relative content may change according to the reaction conditions. Both natural and synthetic indigo are built up from the starting substance indoxyl. Thus, in addition to indigo, the isomers isoindigotin and indirubin are formed in both processes (Figure 1).

In terms of sustainability as a textile dye, natural indigo is equal to synthetic indigo because natural indigo can only be obtained from plants in low concentrations and low purity [21]. There are various, challenging patented purification processes for indigo, and due to the low solubility of indigo and indirubin, there are no known analytical methods for these substances that achieve an accuracy of 2% [22]. In terms of synthesis, biotechnological processes are on the way to sustainably producing indigo [23].

As a gliding layer, the environmental compatibility of synthetic indigo should not be compared to its use as a textile dye, as the toxicologically and ecologically harmful reduction process with sodium dithionite is not required [24,25]. With synthetic instead of natural indigo, a higher yield can be reached, but the byproducts aniline and N-methylaniline are particularly problematic in terms of toxicity [26]. However, it should be noted that aniline is also formed as a decomposition product from natural indigo at high temperatures [27].

Indigo and its isomers form poorly soluble solids in which not only the undesirable isomers, but also the toxic synthetic byproducts aniline and N-methylaniline, are entrapped. Chemical analysis with HPLC requires a solution in DMSO. Separation on a large scale, e.g., to obtain pure E-indigo, is, therefore, not feasible. Similarly, the solubilities in glacial acetic acid, DMF, dichloromethane, chloroform, nitrobenzene, pyridine, and concentrated sulfuric acid are too low for a sustainable purification procedure. The minor physical and chemical differences between the isomers render the separation complex. Therefore, in its main use as a colorant, mixtures of isomers are applied, which differ in hue depending on the relative isomer contents. However, for the application of indigo as a gliding layer, a high content of E-indigo is necessary. For this purpose, the usual separation methods of the isomers, i.e., by sublimation or via reduction and reoxidation, are insufficient. In terms of toxicity, purification must be considered important, due to the observation that mutagenic effects observed in impure indigo decrease with increasing purity [28].

### 2.2. Individual and Collective Properties of Indigo

**Figure 2:**
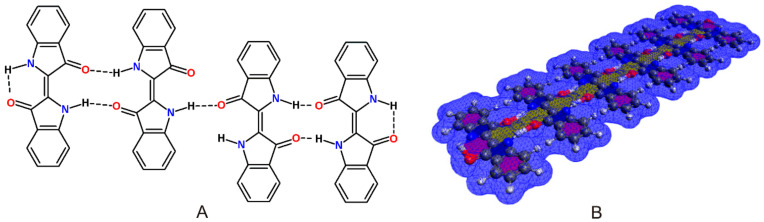
(**A**): E-indigo cluster with intra- and intermolecular hydrogen bonds and the effect of different 2D chirality on the self-assembly. Carbonyl and amino groups without planar hydrogen bonds can form a bond to an upper or a lower layer. (**B**): E-indigo molecules forming a platelet with intermolecular hydrogen bonds (green). The surface is characterized by delocalized electrons in π orbitals.

E-indigo, a dark blue solid, has a density of 1.35 g/cm^3^, sublimes above 300 °C, decomposes above 390 °C, is oxidation resistant, hardly combustible, and has a water solubility below 1 mg/L [29]. The acute toxicities LD50 are 2200 mg/kg (mouse, intraperitoneal) and 32,000 mg/kg (mouse, oral) [30]. The specific properties of indigo are decisively co-determined by the formation of intermolecular hydrogen bonds to build self-assembled supramolecular clusters (Figure 2) [5].

Indigo, as a biogenic substance of plants, is not necessarily harmless, as toxic substances from plants such as the death cap [31] or the monkshood [32] are well known. Plant constituents can also show ecological effects. The walnut tree, for example, secretes growth-inhibiting substances to surrounding plants, and many insects are repelled by the exhalation of its leaves (allelopathy). In addition to the available experimental data [10,33,34,35], it was necessary to determine over 250 parameters to assess the effects of E-indigo on human health and different compartments of the environment. Many important parameters that have not been determined by experiments, or were not available, were predicted using quantitative structure–activity relationship (QSAR) methods [36]. In this assessment, indigo performs very well, both because it is non-reactive and because its functional groups can be attacked enzymatically for biodegradation. Its harmlessness to humans is confirmed by centuries of widespread use as a textile dye. Based on the wealth of available data, it is concluded that indigo has a very low order of both acute and chronic toxicity.

## 3. Indigo as a Gliding Surface

### 3.1. Self-Assembly of Indigo Molecules

E-indigo is particularly stable as a supramolecular cluster with intermolecular hydrogen bonds. Solid lubricants, such as graphite or molybdenum disulfide, reduce friction due to the sheets being able to slide easily on each other. In these crystal structures, the atoms lying in the same sheet are covalently bonded, whereas comparatively weak interactions occur between the stacked sheets. Although a large number of studies on molybdenum disulfide have been published [37,38], quite a few parameters of this lubricant are not well understood. Despite its seemingly significant surface charges and relatively strong van der Waals forces, molybdenum disulfide nanosheets form a hydrophobic and low-friction surface [39]. Indigo does not belong to this class of lubricants, i.e., indigo does not glide on indigo. Supramolecular layers of E-indigo show a high stability, which is due to the π-conjugation in the molecule, as well as to intra- and intermolecular hydrogen bonds. As a monomer, two intramolecular hydrogen bonds between amino and carbonyl groups occupy the most reactive positions for nucleophilic and electrophilic attacks. As dimers, these amino and carbonyl groups of the bound monomers form intermolecular multicentric nonlinear hydrogen bonds in six-membered rings, which protect the same reactive centers and explain the limited reactivity and solubility of indigo. The crystal structure of indigo was refined based on synchrotron data, including the positions of the hydrogen atoms. The >Ν-H···O=C< hydrogen bond can be regarded as bifurcated, with the intermolecular bridge being significantly shorter than the intramolecular bridge [40]. These intermolecular hydrogen bonds were also verified spectroscopically [41].

**Figure 3:**
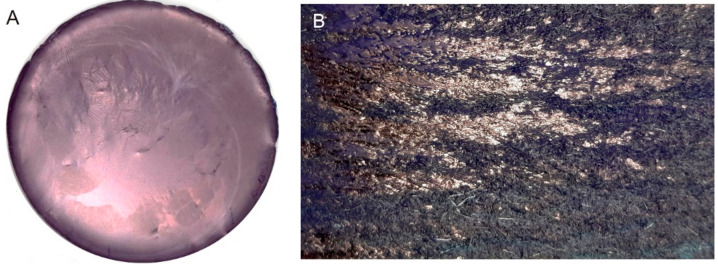
(**A**): indigo layer formed upon filtration. (**B**): pure E-indigo on a friction felt. The metallic luster observed on both photographic images indicates that larger indigo structures have formed by self-assembly from suspension or by friction, respectively [46].

The delocalized electrons of indigo lead to a copper-colored metallic luster, which was already described in 1835, as “Indigmetall” [42] (Figure 3). The delocalization of the electrons forms the basis for applications of indigo layers as semiconductors [9,43]. On liquid surfaces, supramolecular indigo clusters are formed by self-assembly, which only emerge as layers through friction. Experience with polishing shows that some forms of a Beilby layer [44,45] could contribute to improving the slip on these layers.

Three tautomers of E-indigo are possible. The properties of these tautomers have not been described experimentally, but can be estimated using QSAR methods (Table 1) [36]. Indigo can, to a certain extent, adapt to the polarity of the environment, with the diketo form being the most polar and the dienol form being the least polar. It is not known why the water solubility does not follow this trend.

Unlike the paraffins in a gliding layer, the indigo molecules are connected by hydrogen bonds, thus reducing the loss due to abrasion. This is not only important for prolonged use, but also beneficial in terms of leading to a negligible release into the environment. Long multimolecular clusters of E-indigo are ideal for the formation of stable layers, and can only be achieved if the interfering indigo isomers are reduced to minimal levels. Individual multimolecular indigo platelets can form layers on top of each other, connected by π–π stacking [55]. It is known from graphite that the π-bonded bilayers are very stable to temperature and solvents. Analogous formations are also expected for E-indigo [56]. Comparable to the sequence from benzene, naphthalene to hexacene, the lipophilicity increases with an increasing number of E-indigo molecules of the supramolecular structure. The same trends for log(*K*_ow_) and water solubility can be expected from indigo analogues as monomers, dimers, and trimers.

In the process of forming a gliding layer, the indigo molecules self-organize in solution, or can be made to connect by friction. The poor solubility in most solvents limits the use of indigo as a homogeneous gliding layer. Increasing the solubility in chloroform by means of derivatization and subsequent thermolysis at 200 °C can only be justified in the case of expensive components if pure indigo is already present [57]. In addition, the use of chloroform is unsustainable due to its toxicity, and the high temperature of thermolysis is incompatible with many plastics.

**Figure 4:**
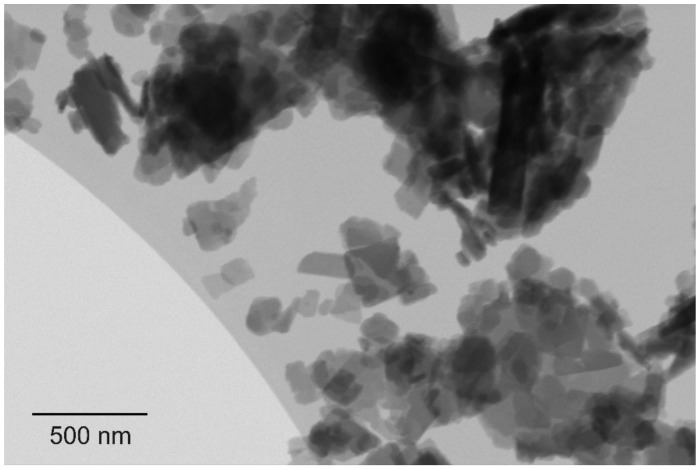
Scanning transmission electron microscopy image, showing primary particles of indigo with platelet morphology [46].

On a surface where the electrons of the bonds between the atoms are hardly accessible, e.g., fluorinated surfaces [58], water molecules only bind weakly. Such surfaces are strongly hydrophobic due to the extreme electronegativity of fluorine. Comparable electric fields can hardly be achieved without fluorine. The strong binding of electrons in aromatic systems, however, points in the same direction, though more weakly. For this reason, E-indigo has poorer gliding properties on water, but better gliding properties on ice and cold snow. The formation of indigo nanoplatelets (Figure 4) is ideal to produce gliding layers. The toxicology of these nanoparticles is insignificant, because once released, the hydrogen bonds are cleaved, and enzymatic degradation starts from the polar end groups [59]. Furthermore, experiments with the runners of a luge have shown that the bonding of an indigo layer to a surface of iron or steel, together with its hydrophobicity, is sufficient to protect against corrosion.

### 3.2. Gliding Properties of Indigo

When investigating regelation, i.e., the phenomenon of ice melting under pressure and refreezing when the pressure is relieved, Michael Faraday suggested, in 1859, that there may be a thin liquid-like layer of nascent ice on the surface [60]. It has often been hypothesized that in skating or skiing, the surfaces are lubricated by a layer of water formed by pressure melting [61], as proven by experiments [62]. Above approximately −16 °C, a tribo-water film is formed by friction on ice and snow [63,64,65], upon which winter sports equipment, such as skates, sleds, skis, or snowboards, glide. Below this temperature, wet friction changes to dry friction, with higher coefficients of friction. The structure, hardness, density, and moisture content of the gliding surface are determined according to the weather, and locally by small-scale influences. It is, therefore, practically impossible to find an optimum gliding surface for all the different conditions, and, moreover, for different loads and speeds [66].

The structures and roughness of the ski base are adapted to the ice and snow properties to minimize gliding friction, e.g., by stone grinding or steel scraping [67]. Effective hydrophobic layers on such surfaces should be thin, in order to not fully cover the structures of the base and, at the same time, exhibit a sufficient hardness to avoid quick abrasion. Additives are required in conventional paraffin waxes in order to increase the hardness and improve the abrasion resistance. In many of the demanding, changing conditions in professional sports, a pure E-indigo layer, as a hydrophobic, thin, hard, solid gliding layer, improves glide, minimizes the uptake of dirt, and reduces abrasion on ice, snow, and water. The adhesion of E-indigo to metal and polyethylene surfaces is superior to paraffin, to the extent that it can be employed as a “primer”. This allows the application of soft waxes for high initial speeds. After abrasion of the soft wax layer, the remaining indigo layer still provides small friction values for the remaining distance.

**Figure 5:**
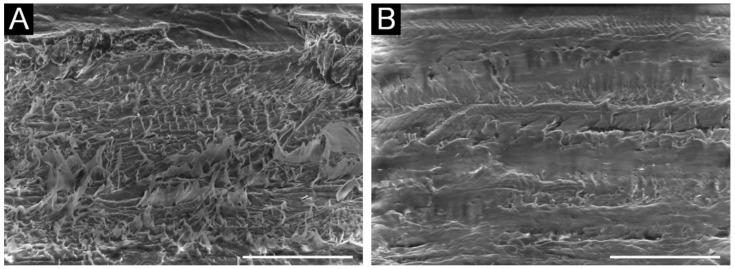
Scanning electron microscopy images showing the roughened surface of the ultra-high-molecular-weight polyethylene (UHMWPE) base before (**A**) and after coating with E-indigo (**B**). The scale bar is 100 µm (52° tilt view) [68].

To form a gliding surface, the entire base surface should be continuously covered with indigo platelets. The self-acting formation of smooth, hydrophobic layers is a condition for low-friction gliding on ice, snow, and water. Alignment of the platelets in the gliding direction decreases the gliding friction. The supramolecular indigo structures can be achieved through self-assembly on the surface of polar liquids, with directed friction and high pressure on the indigo particles (agglomerates), as well as through tempering [5]. The surface structures shown in Figure 5 indicate a smooth coverage with E-indigo when the primary particles have not yet been exposed by tempering, brushing, or polishing. The excellent performance of indigo layers as a gliding surface on snow has been confirmed by field tests (Table 2).

**Figure 6:**
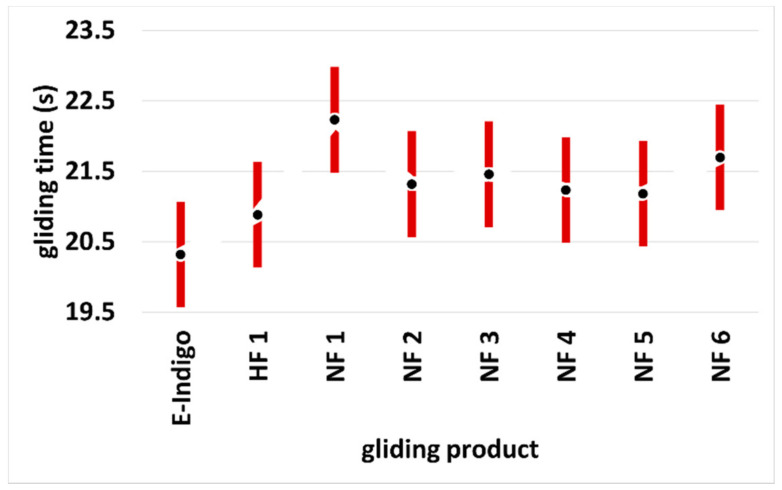
Running times over a measuring section for different ski gliding layers. Tests with calibrated cross-country skis (Lenzerheide, Switzerland, October 2020). The data points show the mean values with the 95% scatter bars. Snow temperature: −8 °C; air humidity: 75%; dry, old snow, grain size 0.5–0.8 mm, star-shaped grains, no dirt (HF: high fluor wax; NF: non-fluorinated wax) [70].

For further development of the gliding properties and the improvement in the abrasion behavior with a single substance as a solid, only the following properties can be changed: the purity, the distribution of the particle sizes, the dispersion medium, the pre-treatment of the base material, the structure of the base material, and the post-treatment of the applied surface (brushing, polishing, heating, etc.). Developments in the last two years have been made with respect to the purity, size distribution, and shape of the particles in the dispersion and dispersants of E-indigo. These improvements have, in two years, reduced the gliding friction on snow (temperature −8 to −2 °C) by a further 2.2%, approximately. Figure 6 shows the promising results of recent field tests, and compares the performance of E-indigo to a high fluor wax and several commercially available non-fluorinated products. The scatter bars show how widely spread the professional field measurements are. They also give clear indications of the value of the individual tests, which can, at best, reflect the local situation and a narrowly defined weather situation over time. This is shown by the different values of the measurements at snow temperatures of −8 °C in Table 2 and Figure 6.

## 4. Conclusions

For hard, continuously bonded gliding layers, multiple applications of several thin layers of pure E-indigo particles with friction in the sliding direction are necessary. Field tests with indigo as a gliding layer on skis, snowboards, and sleds show small coefficients of gliding friction. In addition, abrasion is low, resulting in long operating times. The indigo-coated surfaces protect against corrosion. The preconditions for good indigo glide coatings are high purity (E-indigo), small particle sizes, and sufficient friction to form the self-assembled, supramolecular, thin, hard, and hydrophobic structures. E-indigo is suitable as a supramolecular gliding surface with low gliding friction on ice, snow, and water, and is ecologically compatible as a biogenic material. Experimental and QSAR estimated data show no hazard to human health, thus opening the possibility of new applications in tribology. Future work should focus on sustainable processes for the removal of the synthetic byproducts aniline and N-methylaniline, as well as on the separation of the isomers to obtain high-purity E-indigo.

## Figures and Tables

**Table 1:** E-indigo tautomers with experimental data [10,33,47,48,49,50] and QSAR predicted properties (software: T.E.S.T. [51], EPI-Suite [52], OPERA [53] and VEGA [54]).

	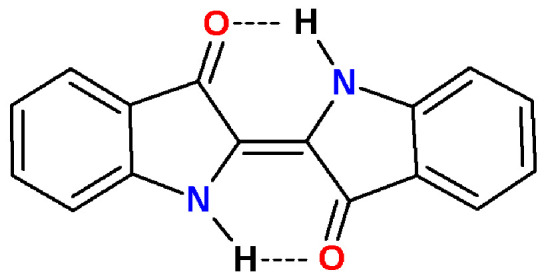	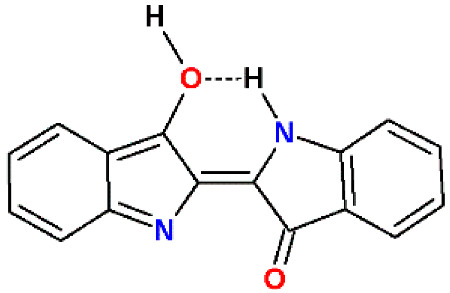	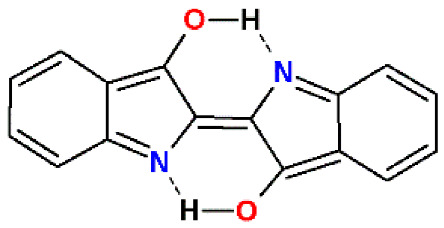
water solubility (mg/L, 25 °C)	exp.: 0.05–2 mg/L pred.: 7.19 ^a^, 13.44 ^b^, 1.0 ^c^	pred.: 59.92 ^a^, 6.36 ^b^, 1.8 ^c^	pred.: 35.68 ^a^, 0.411 ^b^, 8.2 ^c^
log(*K*_ow_)	exp.: 2.7–3.72 pred.: 3.11 ^a^,1.99 ± 0.63 ^d^	pred.: 4.10 ^a^, 2.31 ± 1.50 ^d^	pred.: 5.49 ^a^, 2.53 ± 2.58 ^d^
log(BCF)	exp.: 2.5–4.5 pred.: 5.6 ^a^, 1.5 ^b^, 0.8 ± 0.2 ^c^	pred.: 4.1 ^a^, 1.8 ^b^, 1.6 ± 1.0 ^c^	pred.: 3.8 ^a^, 3.3 ^b^, 2.7 ± 1.7 ^c^
pK_a_	pred.: 9.00 ^c^	pred.: 8.16 ^c^	pred.: 5.17 ^c^

^a^: T.E.S.T.; ^b^: EPI; ^c^: OPERA; ^d^: VEGA.

**Table 2:** Relative time differences per second of running time (diff/s) of comparative field tests with calibrated cross-country skis and different snow temperatures (in parentheses: number of tests with 6 runs each; HF = high fluor and fluor powder, NF = non-fluorinated wax). The mean value at a snow temperature below −8 °C was determined from 4 test series of 4 measurements each. The more negative the value, the smaller the friction [69].

		Snow Temperature		
		Cold <−8 °C	Medium −8 °C to −1 °C	Warm >−1 °C
reference ski (HF)	ski with HF	0.008 (4)	−0.003 (2)	−0.033 (5)
	ski with indigo	0.022 (4)	−0.005 (2)	0.024 (5)
reference ski (NF)	ski with HF	(0)	−0.008 (1)	−0.089 (3)
	ski with indigo	(0)	0.024 (1)	0.057 (3)

## Data Availability

The data presented in this study are available on request from the corresponding authors. QSAR data of indigo and the isomers indirubin and isoindigotin, as well as their tautomers, are available at https://doi.org/10.5281/zenodo.5779028 (accessed on 14 December 2021).
